# Effect of mini-screw-facilitated micro-osteoperforation on the rate of orthodontic tooth movement: a single-center, split-mouth, randomized, controlled trial

**DOI:** 10.1186/s40510-020-00306-8

**Published:** 2020-03-09

**Authors:** Neda Babanouri, Shabnam Ajami, Parisa Salehi

**Affiliations:** Orthodontic Research Center, School of Dentistry, Shiraz University of Medical Sciences, Qom Abad, Ghasrodasht St, Shiraz, 713451836 Iran

**Keywords:** Micro-osteoperforation, Regional acceleratory phenomenon, Rate of orthodontic tooth movement

## Abstract

**Objective:**

The present study aimed to evaluate the effect of MOP over a 3-month period and to determine the influence of the number of perforations on the rate of canine retraction. In addition, the amount of pain and discomfort caused by the MOP method was evaluated.

**Trial design:**

A single-center, split-mouth, triple-blind, randomized, controlled trial was conducted.

**Methods:**

The clinical trial was conducted from December 2018 to July 2019 in the Orthodontic Clinic, Shiraz Dental School. Twenty-eight patients (range from16.3 to 35.2 years) who need fixed orthodontic treatment were recruited and randomly assigned to MOP1 and MOP2 groups. In each patient one side of the mouth worked as a control side which received no MOPs. Four months after first premolars extraction, patients in MOP1 group received 3 MOPs on the buccal surface of alveolar bone in the experimental side to accelerate canine retraction whereas patients in MOP2 group received 3 buccal MOPs and 3 palatal MOPs in the experimental side. The amount of canine retraction was measured every 28 days at three intervals on both sides of the mouth. Pain perception was also measured on the day of MOP procedure and subsequently at 24 h. Randomization was performed using online software RANDOM.ORG; the recruited patients were divided into two parallel groups with a 1:1 allocation ratio then the side of MOPs intervention in each subject was randomly determined with coin tossing. Triple blinding design was employed.

**Results:**

The result of the intra-examiner reliability using ICC was 0.97 (*P* <  0.001), indicating excellent repeatability and reliability of the measurements. The baseline characteristics between the groups were similar (*P* > 0.05). There was a significant difference in the rate of canine retraction between the MOP groups and the contralateral control sides, as well as between the MOP1 and MOP2 groups (*P* <  0.05).

**Conclusion:**

The MOP procedure was effective in accelerating orthodontic tooth movement, although the amount of acceleration was not clinically significant in the case of canine retraction. An increase in the number of MOPs resulted in a significant acceleration of the canine retraction.

**Trial registration:**

The trial was registered 30 November 2018 at the Iranian Registry of Clinical Trials (IRCT20181121041713N1).

## Introduction

The treatment of moderate to severe cases of malocclusion with fixed orthodontic appliances usually takes over 1.5 years [1]. Due to the physical and social discomfort and the prolonged use of fixed appliances, patients tend to avoid such treatment. As a direct result, they opt for alternative methods such as implants or veneers; with less than optimal outcomes [2]. The increased demand for rapid orthodontic treatment has led to the introduction of several methods, which also reduce potential risks of dental and periodontal complications such as external apical root resorption, high levels of dental caries, and subsequent gingivitis and periodontitis [3–5]. Some of the proposed methods are adequate use of brackets, controlling force levels, and relying on less friction bracket systems, photobiomodulation, pharmacological approaches, or low-intensity laser irradiation [6–11]. However, irrespective of the method, the rate of the tooth movement is dictated by the biologic response to the applied orthodontic forces [2, 12]. Increased activity of inflammatory markers (e.g., chemokines and cytokines) in response to orthodontic forces has been reported [13]. Chemokines, directly or indirectly, play an important role in the recruitment of osteoclast precursor cells and cytokines through the prostaglandin E2 and the RANK/RANKL pathways [5, 14]. Therefore, it is logical to presume that increasing the expression of these pathways should accelerate tooth movement [5].

Over the past decade, various surgical methods have been introduced to manipulate biological processes [15–17]. Regional acceleratory phenomenon (RAP) is one of these methods that utilizes bone reaction to a noxious stimulus, which accelerates the bone turnover and reduces regional bone density; leading to transient osteopenia [17–19]. RAP was first used in the Wilckodontics treatment method and has proven effective in accelerating tooth movement [20]. However, this method has the disadvantage of requiring a corticotomy surgical procedure; involving cuts in the cortical bone, raising the split-thickness flap, and bone decortication [3, 20]. Corticotomy techniques have been effective in inducing rapid tooth movement [21]. However, the procedure is relatively invasive as it involves full mucoperiosteal flap elevation, suture, and the associated surgical side effects such as pain, swelling, and slight interdental bone and attached gingiva loss [13, 21]. These are probably the reasons why the procedure is not widely deployed by orthodontists. As a direct result, minimally invasive flapless methods such as corticision, piezocision, and micro-osteoperforation (MOP) have been proposed [2, 6, 22].

Among the surgery-assisted techniques, MOP is a new method to accelerate orthodontic tooth movement through small perforations in the alveolar bone without introducing great surgical trauma. The application of MOP in animal models has shown that small and shallow perforations in the alveolar bone increased the rate of tooth movement without requiring raising flaps, bone graft, or suture [23, 24]. In comparison with the traditional orthodontics, a clinical study over a 1-month period has shown that when perforations are made to one side of the maxilla, the procedure significantly increased the rate of tooth movement by 2.3-fold, causing little pain or discomfort to the patients [2]. In a split-mouth clinical study, over a period of 16 weeks, acceleration in canine retraction by 1.1 mm compared to the contralateral control side was reported [25]. In contrast, another well-designed clinical trial revealed that MOP was not effective in increasing the rate of orthodontic tooth movement over a 3-month study period [26]. A literature review also indicated that there is limited and contradictory evidence on the effectiveness of MOP in accelerating tooth movement in human [6]. It is likely that factors such as small sample size, short follow-up period, potential risk of selection and information bias, and possible conflict of interest have all contributed to such conclusions [6]. Hence, the present split-mouth, triple-blind, randomized, controlled trial was instigated to evaluate the effect of MOP on the rate of orthodontic movement over 3 months and to determine the influence of the number of perforations on the rate of canine retraction.

### Specific objectives and hypotheses

Considering the above, the present study aimed to evaluate the effect of MOP over a 3-month period and to determine the impact of the number of perforations on the rate of canine retraction. In addition, the amount of pain and discomfort caused by the MOP method was evaluated. The null hypothesis was that an increase in the number of perforations (from 3 buccal perforations to a total of 3 buccal and 3 palatal perforations) does not accelerate the rate of canine retraction compared to the non-MOP group and the group receiving fewer perforations.

## Methods and materials

### Trial design

A single-center, split-mouth, triple-blind, randomized, controlled clinical trial was conducted from December 2018 to July 2019 in the Orthodontic Clinic, School of Dentistry, Shiraz University of Medical Sciences, Shiraz, Iran. This study is a three-arm trial with control per MOP method within the same patient. The split-mouth design reduce biologic variables between the experimental group and the contralateral control side. The present study was approved by the Ethics Committee of Shiraz University of Medical Sciences, (code 1396-01-37-15681). The trial was registered at the Iranian Registry of Clinical Trials (IRCT20181121041713N1). The methods used remained unchanged throughout the study.

### Participants, eligibility criteria, and settings

The target population consisted of patients of both sexes who had been referred to the orthodontic clinic of Dental Faculty for fixed orthodontic treatment. The inclusion criteria were age 15–45 years, bilateral class II division 1 malocclusions or class I malocclusion with bimaxillary protrusion; mild or no crowding, no previous orthodontic treatment, no history of systemic disease; no radiographic evidence of bone loss in both sides of the mouth, no history of periodontal therapy or current periodontal disease; probing depth less than 4 mm across the entire dentition, no gingivitis or active carious lesion, gingival index and plaque index value < 1, and being a non-smoker. The exclusion criteria were vertical skeletal discrepancy (62% ˂Jarabak index˂ 65% and 22 ˂ Frankfort mandibular plane angle < 28), systemic disease or medications that affect bone biology, and poor oral hygiene.

Prior to the study, the research goals, intervention methods, and probable risks and benefits were explained to the participants, the confidentiality of any disclosed information was guaranteed, and voluntary participation was emphasized. Written informed consent was obtained from all the participants.

### Sample size calculation

In accordance with a previous study [2], the sample size was determined based on the mean rate of canine retraction (0.67 ± 0.34). The main assumptions were a canine retraction rate of 0.6 mm per month (50% increase), 5% probability of a type I error, and 80% statistical power. Furthermore, on account of using mini-screw as an anchor unit, the amount of canine movement on one side could be considered completely independent from the contralateral side. Accordingly, a sample size of 10 patients per group was calculated. However, to achieve a more accurate estimation and compensate for the potential drop out during the research, the sample size was increased to 14 patients per group. A total of 41 patients were evaluated for eligibility out of which 28 patients fulfilled the inclusion criteria.

### Randomization (random number generation, allocation concealment, implementation)

The patients were equally divided into two experimental groups, namely, the MOP1 and MOP2 group. The allocation of the participants was based on the block randomization method (block length = 4) using the online RANDOM.ORG software. Each random number was placed in a sealed opaque envelope and subsequently, each participant randomly picked one envelope corresponding to either the MOP1 or MOP2 group. For each participant, by tossing a coin, the MOPs intervention was randomly assigned to the right or left side at first premolar extraction sites, while the opposite side served as the split-mouth control.

### Blinding

To ensure both the participants and clinicians were blinded to the clinical trial, a similar number of insertions were created on the buccal surface (in the MOP1 group) or buccal and palatal surfaces (in the MOP2 group) only in the gingival tissue on the control side. MOPs intervention was performed by the first author (NB) such that both the patients and the orthodontist were blinded to the experimental side. No perforation on the cortical bone of the control side was created. In addition, the second author (SHA), responsible for the measurements, and the statistician were blinded to the coding of the study models.

### Intervention

As part of the initial phase, prior to the orthodontic treatment, the periodontal condition of all patients was assessed by the same periodontist. Then, all patients were referred to the same surgeon for the simultaneous extraction of both maxillary first premolars. The leveling and alignment phase of the treatment was initiated with bonding fixed appliances in both arches (Mini Master Brackets, MBT prescription with 0.022-in.; American Orthodontics, USA) by the same orthodontist. Four weekly sequences of 0.014-in., 0.016-in. nickel-titanium (NiTi) (NiTi Memory Wire; American Orthodontics, USA) followed by 0.018-in. and 0.016 × 0.022-in. stainless steel (SS) working archwire (Stainless Steel; American Orthodontics, USA) were performed. Four months after the premolar extraction, the first alginate impressions were taken and immediately poured with plaster. The casts were labeled with the patient’s code and date. Periapical radiographs were taken to estimate the center of resistance of both maxillary canines. The center of resistance of each tooth was determined at one-third of the distance between the alveolar crest and the root apex. A power arm was made using 0.019 × 0.025-in. SS wire, based on the estimated center of rotation, and bonded just mesial to the canine bracket on the buccal surface. The power arm distally extended to the midpoint of the canine to control the potential rotation of the teeth during the retraction. One month prior to the canine retraction initiation, under local anesthesia, 1.6 mm diameter mini-screws were placed bilaterally in the buccal alveolar process between the roots of the second premolar and the first permanent molar and ligated passively to the first molars.

**Fig. 1:**
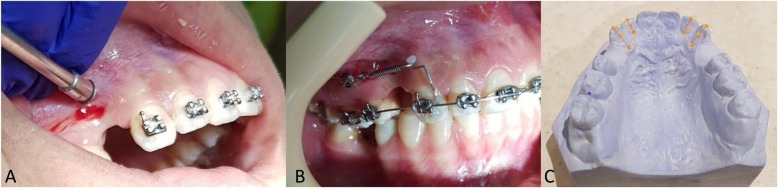
MOP procedure (**a**), closed-coil nickel-titanium spring stretched between the power arm and the mini-screw (**b**), canine retraction was calculated on the casts by measuring the distance between the 2 lines drawing on the midline of canine and lateral incisor, at 3 points: incisal, middle, and cervical thirds of the crowns (**c**)

The clinical MOP procedure was performed by the first author (NB). MOPs were randomly created in the left or right side of the maxilla of each patient by inserting 1.2 mm diameter orthodontic mini-screw (Dual Top Anchor System; Jeil Medical Corporation, Seoul, South Korea) to a depth of 1 mm in the cortical bone under local anesthesia (2% lidocaine with 1:100,000 epinephrine) (Fig. 1a). To ensure a consistent MOP depth of 1 mm, the thickness of the gingival tissue at the mini-screw placement site was measured with an endodontic file and the value was added to the depth of the mini-screw insertion.

Three MOPs were created on the buccal surface of the alveolar process on the experimental side of the MOP1 group. In the MOP2 group, three MOPs on the buccal surface and three MOPs on the palatal surface were created. MOPs were created directly through the alveolar mucosa in the middle of the distance between the distal surface of the canine and the mesial surface of the second premolar at the extraction site, in the vertical direction and 3 mm apart. The first MOP was located 5 mm away from the free gingival margin. Concurrently, local anesthesia was applied on the control sides and extremely shallow insertions were made corresponding to those of the experimental sides, but only in the gingival tissue. For the canine retraction, a calibrated 150 g NiTi closed coil spring (American Orthodontics, USA) was used, which was connected from a temporary anchorage device to a power arm on the canine surface to induce bodily movement (Fig. 1b). The participants were instructed to avoid the use of anti-inflammatory medication and only take acetaminophen if needed. At each visit, the force produced by the coil was checked and the appliances were monitored for any deformation or change in position due to chewing.

### Outcomes (primary and secondary) and any changes after trial commencement

The primary outcome was the rate of canine movement measured indirectly on the plaster models using a digital caliper (accuracy 0.01 mm). Alginate impressions were taken immediately before canine retraction, and at three 28-day intervals (T1, T2, and T3) after canine retraction to monitor the movement rate. The total follow-up period was 84 days from the initiation of the canine retraction. Vertical lines were drawn on the cast over the palatal surface of the canine and lateral incisor from the middle of the incisal edge to the middle of the cervical line (Fig. 1c). Before and after canine retraction, the distance between the canine and the lateral incisor was assessed at three points (incisal, middle, and cervical thirds of the crowns) at different time intervals (T1, T2, and T3).

Secondary outcome: to assess the amount of pain associated with the MOPs, the patients were asked to mark the level of pain and discomfort on each side of the maxilla, both on the day of canine retraction and 24 h later, using a visual analog scale (VAS). VAS is a 10 cm line scaled from 0 (no pain) to 10 (the worst possible pain). The response from the participants was obtained during the first visit after the MOP procedure. There were no changes to the outcome measures after the trial initiation.

### Interim analysis and stopping guidelines

Not applicable.

### Statistical analysis

Data analysis was performed using SPSS software (Version 15.0, SPSS Inc., Chicago, IL, USA). The homogeneity of the groups in terms of sex, age, and cephalometric parameters was assessed using the chi-square test and *t* test. A mixed model analysis with a 3-model categorical predictor (MOP1, MOP2, control) was used to compare the amount of tooth movement and pain perception between the groups and also assess their changes in each group with respect to the time. The level of significance was set to *P* <  0.05 for all statistical analyses.

## Results

### Participant flow

**Fig. 2:**
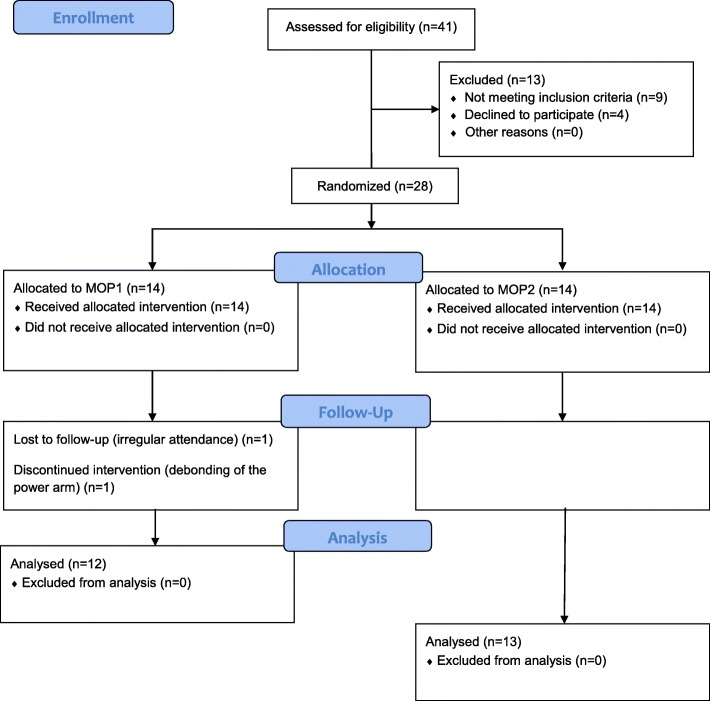
CONSORT flow diagram displaying the progress of all participants through the trial

Following the MOP procedure, two patients in the MOP1 group and one patient in the MOP2 group were excluded from the study due to either debonding of the power arm on the canine or irregular attendance (Fig. 2).

### Baseline data

The final sample size was reduced to 25 patients, 14 women and 11 men, with an age range from 16.3 to 35.2 years. Information regarding sex, age, and morphological characteristics of the patients are outlined in Table 1.

**Table 1:** Sex, age, and cephalometric characteristics of the current sample

Groups	Sex (*n*, %)		Age	Jarabak index	ANB	FMA
	Female	Male	Mean ± SD			
MOP1	7 (58.3)	5 (41.7)	26.08 ± 9.15	65.22 ± 1.58	5.33 ± 1.69	25.25 ± 1.48
MOP2	7 (53.8)	6 (46.2)	25.31 ± 9.03	25.31 ± 9.03	5.23 ± 1.61	24.69 ± 0.85
*P* value^*^	–	–	0.83	0.69	0.87	0.25

^*^Paired *t* test

### Error of the method

All measurements on the study models were done by one examiner (SHA). To evaluate the intra-examiner reliability, 15 study models were randomly chosen and measured twice within a 2-week interval. The intra-examiner reliability was assessed using the intraclass correlation coefficient (ICC). The result of the intra-examiner reliability using ICC was 0.97 (*P* <  0.001), indicating excellent repeatability and reliability of the measurements.

### Outcomes

#### Primary outcome

Deceptive data including mean and standard deviation of the amount of canine retraction and pain perception in three study groups are summarized in Table 2. Obviously, the mean value of the canine movement was greater in MOP2 than other study groups.

**Table 2:** Canine movement values (mm) and pain values (VAS results) of the MOP1, MOP2, and control groups. Data are expressed as mean ± SD

	Time	Group		
		MOP1	MOP2	Control
Incisal	Day 28	0.94 ± 0.31	1.21 ± 0.20	0.64 ± 0.12
	Day 56	0.86 ± 0.19	1.14 ± 0.18	0.66 ± 0.16
	Day 84	0.76 ± 0.10	1.0 ± 0.15	0.73 ± 0.12
Middle	Day 28	0.88 ± 0.26	1.18 ± 0.20	0.61 ± 0.12
	Day 56	0.86 ± 0.19	1.11 ± 0.18	0.62 ± 0.12
	Day 84	0.76 ± 0.10	1.08 ± 0.15	0.68 ± 0.12
Cervical	Day 28	0.83 ± 0.24	1.16 ± 0.19	0.59 ± 0.11
	Day 56	0.79 ± 0.16	1.09 ± 0.19	0.61 ± 0.12
	Day 84	0.66 ± 0.12	1.08 ± 0.21	0.66 ± 0.10
Pain	T0	3.66 ± 2.34	3.61 ± 2.59	2.96 ± 2.26
	T1	1.75 ± 2.22	1.53 ± 1.85	1.40 ± 1.50

*T0* on the day of MOP, *T1* 24 h after MOP procedure

**Fig. 3:**
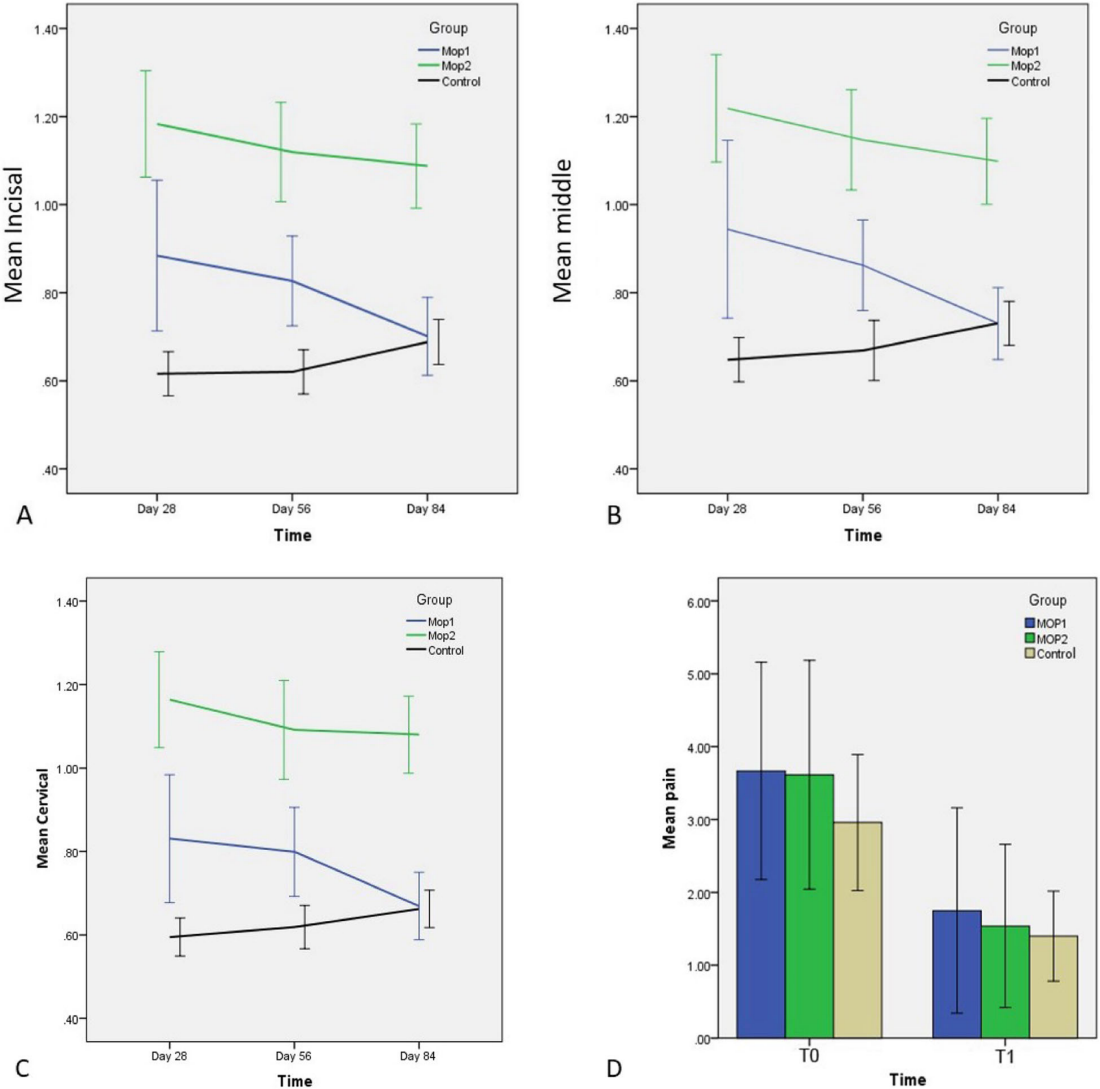
Comparison of mean values of the canine movement among three study groups over the time at the incisal, middle, and cervical points (**a**, **b**, **c** respectively). Comparison of the mean pain scores on VAS among the three study groups at two time intervals (**d**). T0, on the day of MOP; T1, 24 h after MOP procedure

Mixed model results showed that interaction between the group and time as well as the main effect of them were significant in the incisal, middle, and cervical areas. The *P* value was *P* <  0.001, *P* < 0.001, and *P* = 0.008 in the incisal point for the interaction, group, and time, respectively. In the middle area, *P* value was *P* < 0.001, *P* < 0.001, and *P* = 0.010. The value in the cervical point was *P* < 0.001, *P* < 0.001, and *P* = 0.027 for the interaction, group, and time, respectively. So, an interaction effect has been found between the group and time. As the main results were affected by the interaction effect, the separate results according to the main effect were provided which were summarized in Table 3. Model coefficient and its 95% confidence interval with *P* value were also reported in this table. The results showed that there was no significant difference in canine movement between the MOP1 and the control group at T3 (day 84) and the results were the same for all three points on the teeth (incisal, middle, cervical). In the MOP2 group, the amount of tooth movement was significantly greater than the control group during the entire study period as well as for all three points on the teeth (incisal, middle, cervical) (Fig. 3a–c).

**Table 3:** Model coefficient, its 95% confidence interval and *P* value of the canine movement

Area	Time	Group											
		MOP1				MOP2				Control			
		*B*	95% CI of B		*P*	*B*	95% CI of B		*P*	*B*	95% CI of B		*P*
Incisal	Day 28	Ref											
	Day 56	− 0.08	− 0.21	0.05	0.209	− 0.07	− 0.19	0.05	0.245	0.02	− 0.03	0.07	0.471
	Day 84	− 0.21	− 0.37	− 0.05	0.012	− 0.12	− 0.22	− 0.01	0.034	0.08	0.03	0.13	0.002
Middle	Day 28	Ref											
	Day 56	− 0.05	− 0.16	0.05	0.269	− 0.06	− 0.18	0.05	0.282	0.00	− 0.05	0.06	0.876
	Day 84	− 0.18	− 0.29	− 0.06	0.005	− 0.09	− 0.19	0.00	0.06	0.07	0.02	0.11	0.004
Cervical	Day 28	Ref											
	Day 56	− 0.03	− 0.14	0.08	0.548	− 0.07	− 0.18	0.04	0.205	0.02	− 0.01	0.06	0.255
	Day 84	− 0.16	− 0.27	− 0.04	0.011	− 0.08	− 0.18	0.01	0.099	0.06	0.02	0.10	0.002
	Group	Time											
		Day 28				Day 56				Day 84			
		*B*	95% CI of B		*P*	*B*	95% CI of B		*P*	*B*	95% CI of B		*P*
Incisal	Control	Ref											
	MOP1	0.29	0.15	0.44	< 0.001	0.19	0.07	0.31	0.002	− 0.00	− 0.09	0.09	0.993
	MOP2	0.57	0.43	0.71	< 0.001	0.47	0.36	0.59	< 0.001	0.36	0.27	0.45	< 0.001
Middle	Control	Ref											
	MOP1	0.26	0.13	0.39	< 0.001	0.20	0.10	0.31	< 0.001	0.01	−0.08	0.10	0.790
	MOP2	0.56	0.43	0.69	< 0.001	0.49	0.39	0.60	< 0.001	0.39	0.30	0.49	< 0.001
Cervical	Control	Ref											
	MOP1	0.23	0.11	0.35	< 0.001	0.18	0.06	0.29	0.002	0.00	−0.08	0.09	0.879
	MOP2	0.56	0.45	0.68	< 0.001	0.47	0.36	0.58	< 0.001	0.41	0.33	0.50	< 0.001

*B* model coefficient, *CI* confidence interval, *P P* value, *ref.* reference

Our results also showed that there was no significant difference in amounts of tooth movement between the incisal and cervical points of the canine (*P* > 0.05). Similar results were obtained for all study groups and at all time intervals.

#### Secondary outcome

Group effect and time* group interaction effect was not significant in pain but the main effect of time was significant (*P* = 0.864, *P* = 0.536, and *P* < 0.001, respectively). In all study groups, the pain significantly decreased after 24 h. (Fig. 3d).

### Harms

No harm or negative untoward effects were observed during the trial.

## Discussion

### Main findings in the context of the existing evidence

The MOP procedure, a minimally invasive and flapless surgical method to accelerate orthodontic tooth movement, has in recent years attracted the attention of orthodontists. However, there is limited and contradictory evidence on the efficacy of this procedure [6, 13].

The present study aimed to evaluate the effect of MOP on the rate of orthodontic tooth movement during the canine retraction over a 3-month period. In addition, we investigated whether an increase in the number of MOPs could increase the rate of canine retraction. A total of 28 patients participated in the present single-center, split-mouth, triple-blind, randomized, controlled clinical trial. The participants were randomly assigned to two equal groups. To evaluate the effect of MOP on the rate of orthodontic tooth movement during the canine retraction, we utilized the split-mouth study design. The main advantage of this design is the reduced biological variables and therefore requiring a lower sample size [27].

In line with previous studies, our results showed that, except for the T3 time interval in the MOP1 group, there was a significant increase in the rate of canine retraction compared to the contralateral control side in both groups at all time intervals [2, 23–25]. However, another split-mouth clinical trial study found no significant effect of MOP on the rate of tooth movement over a 3-month period [26]. Compared to the present study, the main difference was in performing canine retraction using a 0.019 × 0.025-in. SS wire on a 0.022-in. slot bracket, whereas we used a 0.016 × 0.022-in. SS wire on a 0.022-in. slot bracket. Although wire ligation was used to reduce the friction, a possible higher level of friction in that study could explain such contradictory findings. Moreover, different reference points were used to measure the amount of canine movement. The researchers argued that the maxillary lateral incisors cannot serve as a reliable reference point due to their potential movement during the canine retraction. Whereas, we used these teeth as a reference since we believed that the ligation of the four incisors could prevent the potential undesired movement.

There was a significant increase in the rate of canine retraction in the MOP2 group compared to the MOP1 group at all time intervals. This finding may be attributed to the greater surgical trauma that stimulated a higher expression of inflammatory markers and osteoclast activity, which in turn increased the rate of tooth movement [19, 28, 29]. Interestingly, there was no significant difference between the experimental side and contralateral control group at T3 time interval in the MOP1 group. This could be explained by the transient nature of RAP which weakened over time [19, 21].

As the first study on MOPs with triple-blind design, we aimed to reduce the risk of bias and rule out confounding variables. A triple-blind design can prevent any risk of information bias and conflict of interest. Occlusal force is one of such confounding factors that can affect the rate of orthodontic tooth movement. Patients may avoid chewing with the MOP side; therefore, blinding the participants probably prevented any undesired alteration of their habitual occlusion. To eliminate the effect of occlusal force among the participants, those with a similar growth pattern in the vertical dimension were included in the study. In addition, primary leveling and alignment reduced occlusal interference during the canine retraction stage. Another confounding variable that can affect the rate of orthodontic movement is age; the rate of tooth movement is faster in younger patients [30–32]. This effect has been attributed to the rate of osteoclast activity and recruitment as well as bone density [31, 32]. To rule out the effect of this factor on the rate of tooth movement, only patients older than 15 years of age were enrolled in the study and we ensured that both study groups had the same average age.

Animal studies have demonstrated that sex hormones could affect the rate of orthodontic movement [33, 34]. However, human studies have not shown any such significant difference between men and women [31]. In previous human studies on MOPs, there were more female than male participants [2, 25, 26]. Whereas in the present study, we recruited an almost equal number of men and women to eliminate the potential effect of sex on the rate of orthodontic tooth movement. As a direct result, there was no significant difference in sex between the study groups.

The type of tooth movement was another factor that could affect the tooth movement rate. In the present study, we made an effort to reduce the degree of tipping of canine through the use of the power arm and in applying the acting force at the center of resistance of the canine. However, complete bodily movement was not achieved and an insignificant degree of tipping was involved in both study groups. A previous study argued that the tipping movement reported in MOP studies resulted in a false-positive increase in the rate of tooth movement [26]. However, we found no significant difference in the amount of tipping between the groups, and thus it could not be responsible for the accelerated tooth movement.

One of the main claimed benefits of the MOP procedure is shorter orthodontic treatment duration through the acceleration of tooth movement. Orthodontic treatment period has been affected by several factors such as orthodontist’s proficiency and experience, appropriate treatment planning and biomechanics, patients cooperation and age, and of course the rate of tooth movement. In the present study, the achieved overall increase in canine retraction using MOP was 0.49 mm and 0.15 mm in the middle point of the crowns of the MOP2 and MOP1 groups, respectively. This level of acceleration is probably not clinically significant enough to justify its use as a routine intervention during orthodontic treatments. This is particularly the case considering that in routine clinical practice, the extraction of the premolar just before the canine retraction can stimulate RAP and increase the rate of orthodontic movement. Nonetheless, the benefits from the additional acceleration are valuable enough bearing in mind the challenges associated with some orthodontic tooth movements; e.g., space closure in the old extraction site or uprighting of the posterior teeth. Accelerating tooth movement by MOP procedure could expand our treatment possibilities for example potential decrease in the anchorage value of the target teeth, which could be especially valuable in the case of differential expansion or intrusion. Moreover, temporary osteopenia as a result of RAP potentially changes the center of resistance of the tooth and also the optimum force level. Further studies on these topics are recommended.

One of the unanswered questions about MOP is the frequency of application. Based on the results of the presents study it is wise to use MOPs every 56 days in buccal MOPs and at least every 84 days in the case of buccal and palatal MOPs.

In line with previous studies, our results did not show any difference in pain perception among three study groups [2, 25, 26]. However, since the retraction force was applied immediately after the MOP procedure, it is highly probable that the patients could not differentiate the orthodontic pain from the MOP pain. In addition, since gingival insertions in the contralateral control side were created for blinding purposes, and the result of the VAS pain scores between the two sides is questionable.

### Limitations

As the main limitations of the present study, the inflammatory markers were not assessed, the follow-up period did not continue until complete canine retraction, and the sample size was probably too small for the pain assessment. The degree of tooth rotation has been not measured in this study, although tried has been made to control the amount of rotation by distally extended power arm. In addition, other potential side effects of orthodontic tooth movement and MOPs such as root resorption and crestal bone resorption were not evaluated.

#### Generalizability

The generalizability of our findings might be limited since the study was a single-center trial conducted by one clinician over a short period of time and also the potential risk of carrying across effects for pain assessment.

## Conclusion

The findings of the present study indicated that the MOP interventions were effective in accelerating tooth movement over a period of 3 months. In addition, an increase in the number of MOPs (from 3 to 6) resulted in a significant acceleration of the canine retraction. However, the increased tooth movement following MOPs was not clinically significant. Considering the limitations of the study, there was no increase in the level of pain and discomfort due to the MOP procedure.

## Data Availability

The authors agree with sharing, copying, and modifying the data used in this article, even for commercial purposes, so long as appropriate credit is given, and possible changes are indicated.
